# An easily overlooked cause of haemoptysis and heart failure; anomalous systemic arterial supply to normal lung

**DOI:** 10.1259/bjrcr.20190089

**Published:** 2020-09-29

**Authors:** Katherine Luke, Rhys Thomas, Gareth Tudor

**Affiliations:** 1 Department of Radiology, University Hospital of Wales, Cardiff and Vale University Health Board, Wales, UK; 2 Department of Radiology, Princess of Wales Hospital, Cwm Taf Morgannwg University Health Board, Wales, UK

## Abstract

Systemic arterial supply to a segment of normal lung is rare.^1^ Usually the anomalous systemic artery arises from the descending aorta, although it can arise from other sites including the coeliac axis.^1–3^ Case reports documenting an anomalous artery to normal lung from the coeliac axis are few. However, in these cases the patients were being investigated for respiratory symptoms and all were under the age of 50. In our case, we describe a rare case of anomalous systemic arterial supply arising from the coeliac axis to the right lower lobe, in the absence of abnormal bronchial connection or parenchymal disease in an asymptomatic patient. The anomalous arterial supply was an incidental finding on CT. The literature suggests surgical treatment to prevent symptoms of haemoptysis or congestive cardiac failure, but it is unclear from current evidence whether this is indicated in an asymptomatic patient.

## Clinical presentation and investigations

**Figure 1.**
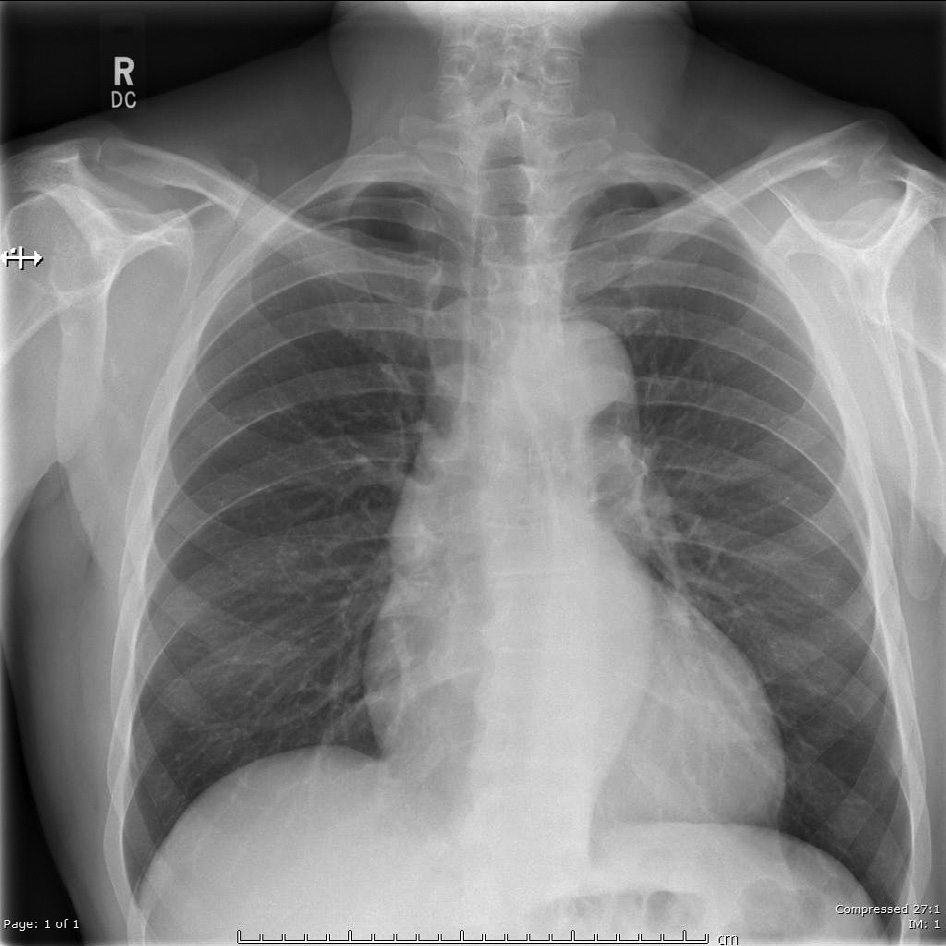
The PA chest radiograph appeared normal, including the pulmonary vasculature. There was no evidence of a linear opacity, which was often reported in the previous cases. PA, posteroanterior

A 53-year-old male marathon runner was being investigated by the cardiologists for hypertension. Past medical history included typical atrial flutter, diverticular disease and a benign polyp in the sigmoid colon. There were no reported respiratory symptoms or any history of respiratory disease. The posteroanterior (PA) chest radiograph was normal, including the appearance of the pulmonary vasculature and lung parenchyma (Figure 1). The echocardiogram reported mild left atrial dilatation and a dilated aortic root, measuring up to 49 mm in diameter at the Sinus of Valsalva, consequently a CT aortogram was arranged.

**Figure 2.**
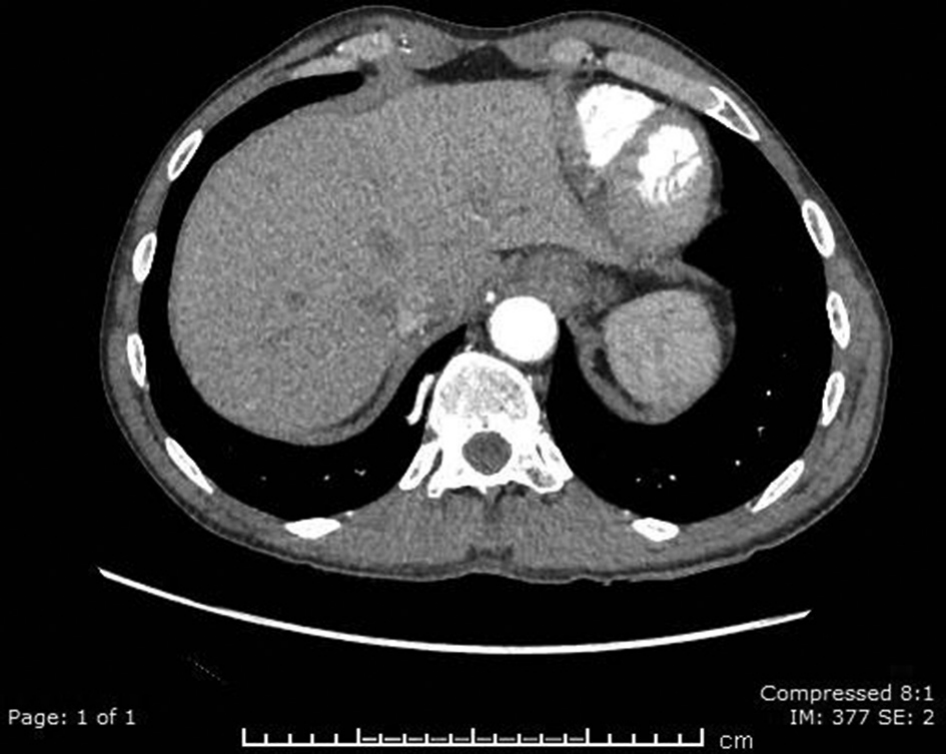
This CT aortogram image demonstrates the branch of the coeliac artery as it traverses the medial basal segment of the right lower lobe.

**Figure 3.**
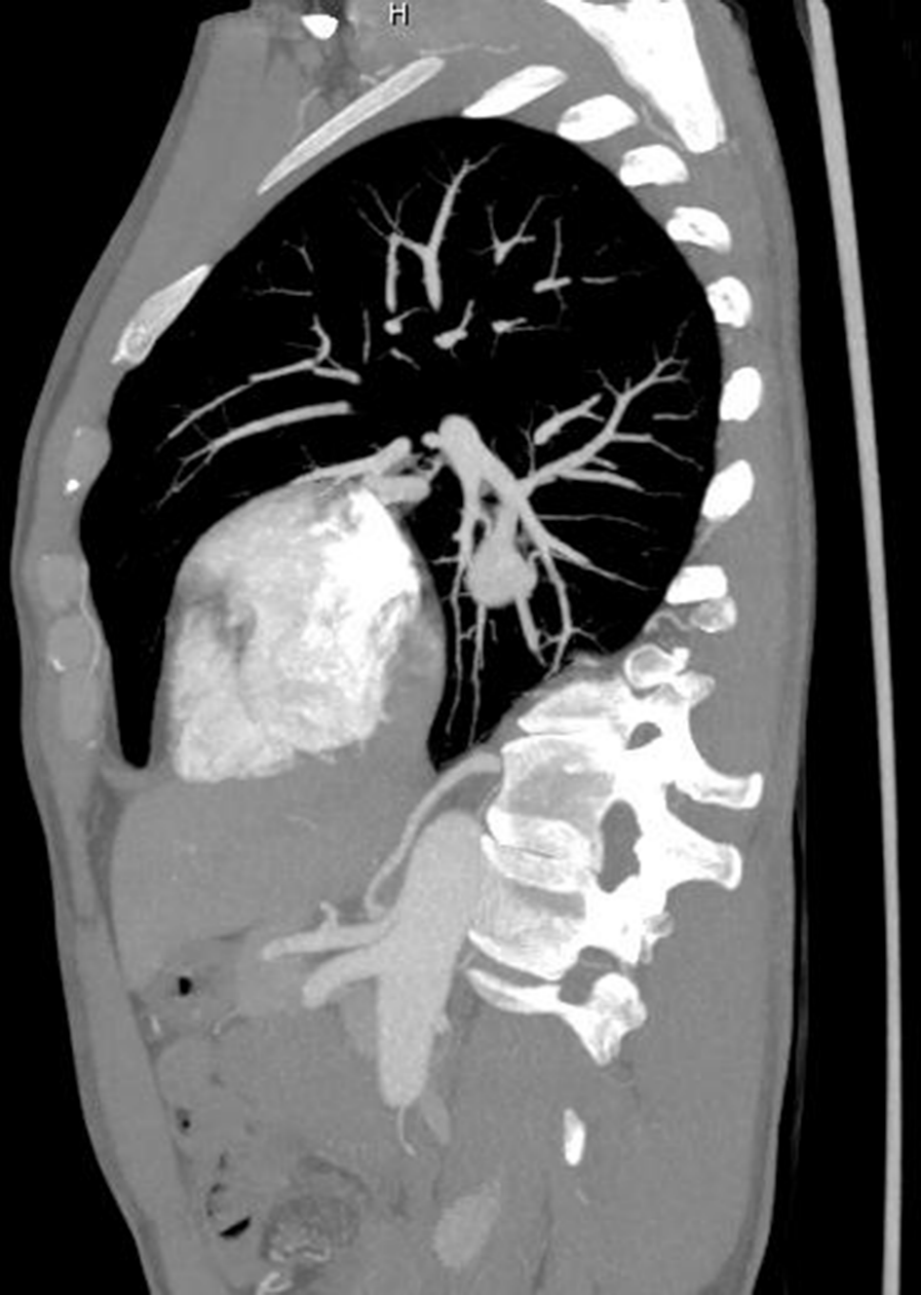
On this sagittal view of the CT aortogram, the branch of the coeliac axis can be seen ascending towards the lungs.

**Figure 4.**
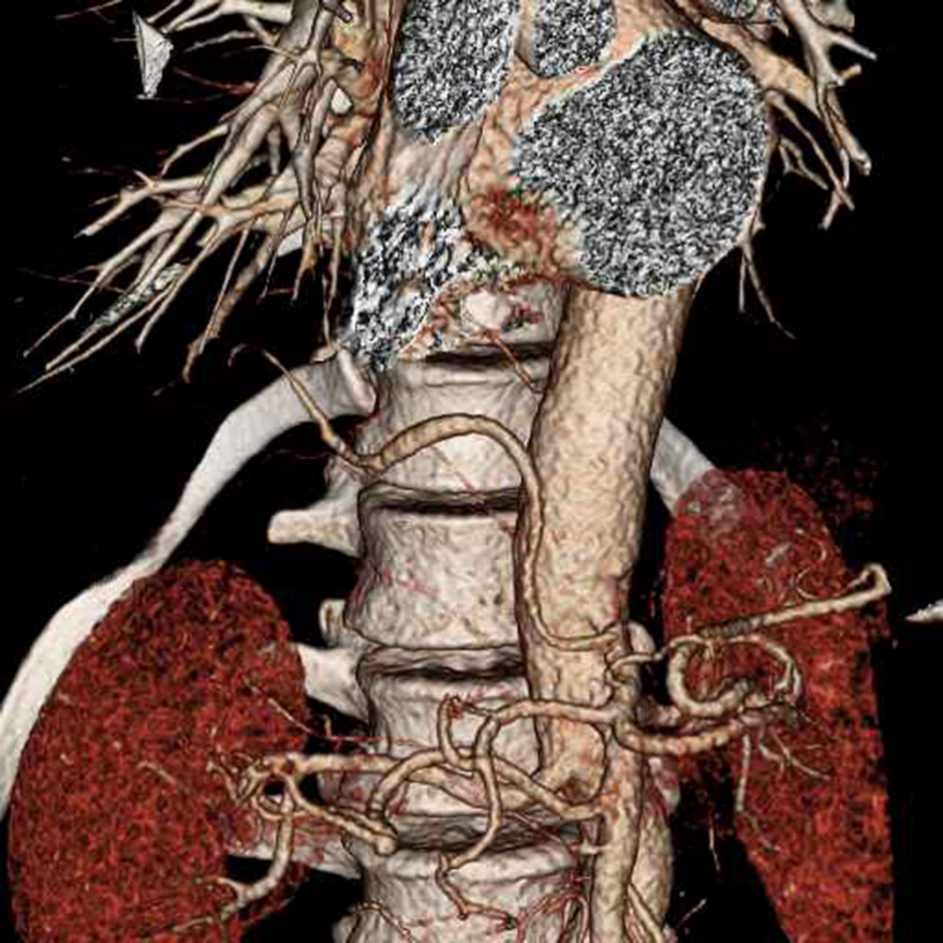
Both the normal tracheobronchial tree and the anomalous artery to the medial basal segment to the right lower lobe are appreciated on this reformatted image CT.

**Figure 5.**
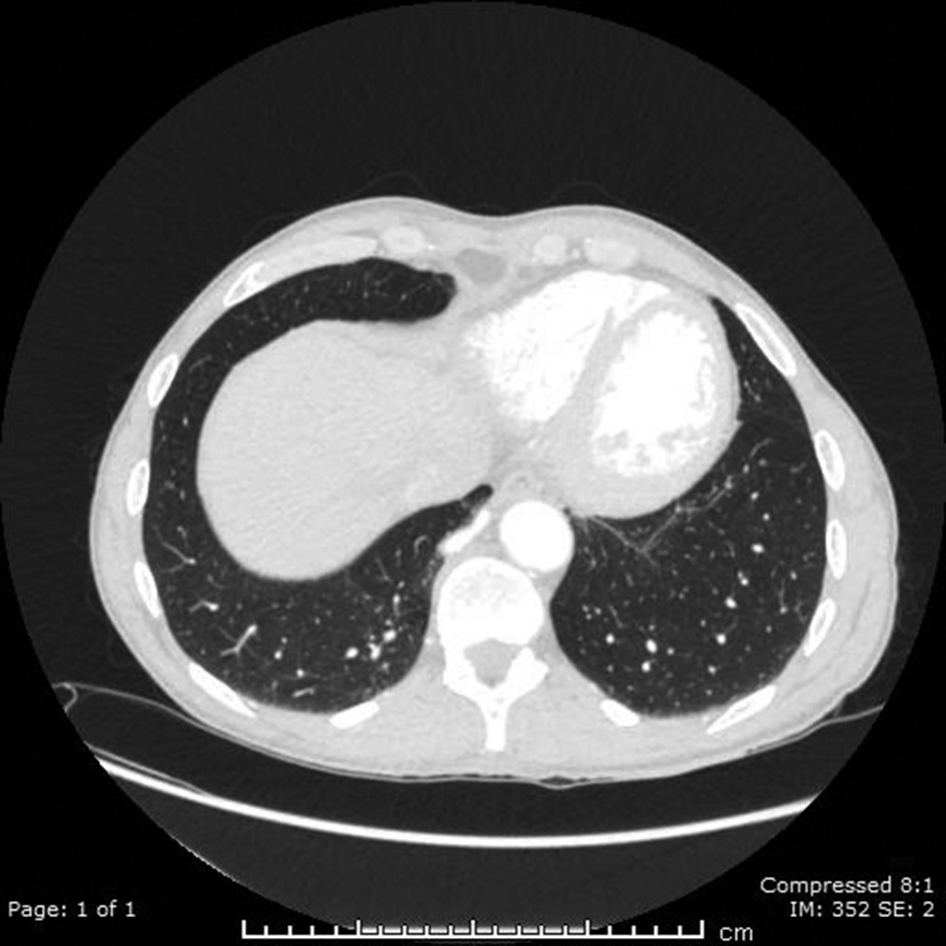
The normal parenchyma of the medial basal segment can be visualized on lung windows of the CT aortogram.

A CT angiogram of the aorta was performed on a GE Revolution, with 100 ml of omnipaque 350 at a rate of 4 ml/s, acquiring 0.65 mm helical slices from above the arch of the aorta to the level of the acetabulum. This revealed that a branch of the coeliac axis supplied the medial basal segment of the right lower lobe (Figures 2 and 3). Normal pulmonary venous drainage into the right inferior pulmonary vein was present and the pulmonary artery branch to the medial basal segment was attenuated. The right lower lobe tracheobronchial tree was normal (Figure 4), as were the appearances of the lung parenchyma (Figure 5). The patient was also noted to have two left renal arteries. A CT colonoscopy had been performed a number of months previously demonstrating diverticular disease and a polyp in the sigmoid colon. An incidental "anomalous vascularity" in the right lower lobe had been reported on this study. Given this was a portal venous phase study with only the lung bases imaged the anomalous artery, pulmonary venous drainage and small medial basal pulmonary artery could not be fully appreciated.

## Discussion

Bronchopulmonary sequestration was first described in 1946 to define abnormal lung tissue which lacked communication with the bronchial tree with an arterial supply arising from the aorta.^2^ Subsequently, it has been debated as to whether anomalous systemic artery supply to normal lung should fall within the "sequestration spectrum." There have been attempts to improve nomenclature, to better reflect the bronchial connection, arterial supply and venous drainage, however the complexity of these abnormalities makes it difficult.^2^

Systemic arterial supply to a portion of the lung with a normal connection to the bronchial tree is rare.^1^ It can be acquired, where chronic inflammation or infection causes hypertrophy of normal systemic arteries, or congenital.^4^ Congenital anomalous systemic arterial supply favours the left lung and basal segments.^2,4^ Usually, the anomalous artery arises from the descending thoracic aorta, however less frequently it may arise from the abdominal aorta or other vessels including the coeliac axis.^2,3^

Patients may be asymptomatic or present with haemoptysis, a murmur or cardiac failure.^2,4–6^ On a PA chest radiograph, the anomalous artery may be seen as a lung opacity.^4–6^ Contrast-enhanced CT is the preferred imaging method as it non-invasive and able to assesses the anomalous systemic artery, pulmonary arteries, tracheobronchial tree and lung parenchyma.^4,5^ The pulmonary artery is usually absent distal to the systemic artery, although less commonly there is both pulmonary artery and systemic artery supply to the same segment.^5^

Our literature review revealed a handful of cases whereby a segment of the lung, with normal bronchial connection, was supplied by the coeliac axis. Campbell et al cite a case report from Batts in 1939 whereby a branch of the coeliac artery supplied the posteroinferior lower lobe in a 40-year-old man,^3^ however there were no further details supplied regarding the case. In 1968, Painter et al described a 24-year-old male who presented with haemoptysis, a “linear shadow” on the chest radiograph and a normal bronchoscopy.^7^ A subsequent pulmonary angiogram demonstrated that the coeliac artery gave rise to a branch which entered the right lower lobe. This artery was then ligated and the anterior and lateral basal segment resected.^7^

More recently, it has been possible to demonstrate these abnormalities on CT. In 1982, a 26-year-old patient was initially treated for pulmonary embolus after presenting with recurrent haemoptysis and a ventilation–perfusion mismatch.^1^ The patient represented with dyspnoea, recurrent fever, cough and haemoptysis.^1^ “Patchy consolidation” and a “band-like density” at the right heart border were described on chest radiography, followed by a normal tracheobronchial tree on bronchoscopy.^1^ An aortogram and pulmonary arteriography demonstrated a large anomalous artery arising from the coeliac axis which supplied the right lower lobe, normal pulmonary venous drainage and the absence of normal right basal pulmonary artery branches.^1^ The anomalous artery was demonstrated on CT and the patient then treated with thoracotomy and ligation of anomalous vessel.^1^

Subsequently, a breathless 34-year-old with a “tubular shadow” on the chest radiograph was found on CT to have a branch of the coeliac trunk and a small segmental pulmonary artery supplying the right lower lobe and normal pulmonary venous drainage.^8^ The patient declined surgery and was asymptomatic at 6 months.^8^

In 2010, the case of a 47-year-old with haemoptysis and abnormal linear opacities on the chest radiograph was reported.^5^ Following a normal bronchoscopy, a HRCT revealed dilated vessels and ground glass change in the left lower lobe. This prompted CT angiography which demonstrated that the left lower lobe was supplied by a systemic artery arising from the coeliac axis.^5^ The left basal pulmonary artery was present but small in calibre compared to the right, whilst pulmonary venous drainage was again normal. The patient declined surgery and was being followed-up.^5^

A 27-year-old patient presenting with haemoptysis and following a normal chest radiograph was found on CT angiography to have an aberrant supply to an otherwise normal right middle lobe.^6^ Digital subtraction angiography (DSA) confirmed the artery arose from the coeliac axis and the vessel was embolized using glue.^6^

Although patients usually have minimal symptoms, traditionally treatment has been recommended as there is a risk that haemoptysis or congestive cardiac failure will develop over time.^9^ Multiple treatments have been described including lobectomy, segmentectomy, endovascular treatment or anastomosis of the systemic artery and pulmonary artery.^5^ There are no trials directly comparing treatment outcomes.

Isolated systemic supply to the lung is more common than dual supply from both a systemic artery and pulmonary artery.^5^ Lobectomy or segmentectomy with ligation of anomalous systemic artery has been favoured to date, mostly due to concerns that ligation of the systemic artery or endovascular treatment would result in lung infarction^2^ and that in adults there will already be changes in the anomalous systemic artery and lung parenchyma.^9^ Thorascopic surgery has been reported to reduce pain and scarring but can increase the difficulty of managing massive haemoptysis.^9^ Transarterial embolization has been suggested as an alternative option to lobectomy or segmentectomy, even in cases with only isolated systemic artery supply, as a case series demonstrated that the incidence of pulmonary infarction is low and there were no severe consequences on long-term follow-up.^10^ Although treatment has been conventionally recommended, there are reports of patients electing for monitoring.^5,8^ Without long-term follow-up of these patients and given the rarity and variability of the disease the best treatment option is debateable.

Whilst some of the radiological aspects of our case have already been described in the cases above, we present a unique case. Collectively, the aforementioned cases generally describe a young cohort of patients presenting with respiratory symptoms, which usually have an abnormality on the initial chest radiograph and each having had a surgical or endovascular treatment option offered to them. We describe a case of an older—53-year-old—asymptomatic patient, with a normal chest radiograph and an incidental discovery of an anomalous systemic arterial supply from the coeliac axis to normal lung. Whilst the anomaly itself is important to identify, it was not felt that the patient was adversely affected by this and has been treated conservatively.

## Conclusion

Symptomatic cases of anomalous systemic arterial supply to the lung are likely to require treatment. The most likely presentation in symptomatic adults is haemoptysis,^4–6^ which is believed to be due to the higher systemic arterial pressure compared with the normal lower pulmonary arterial pressures.^6^ Some literature recommends treatment in asymptomatic patients to avoid future complications,^9^ however the rarity of such anomalies has precluded any randomized controlled studies, limiting the evidence. There are several treatment options described in the literature, with lobectomy or segmentectomy often favoured in adults^9^ and transarterial embolization proposed as a possible alternative.^10^ Treatment, therefore, requires careful discussion between the clinical team and patient, particularly in asymptomatic individuals.

In our case, the incidental discovery of the anomalous vessel in an asymptomatic 53-year-old gentleman raises the question whether treatment would provide any significant benefit. Nonetheless, knowledge of such anomalous arterial supply to the lung is important should the patient develop symptoms or require future intervention, particularly endovascular procedures involving the aorta and mesenteric vessels.

## Learning points

- Anomalous systemic arterial supply to the lung is a rare cause of haemoptysis or cardiac failure in adults.
- Arterial phase CT imaging is recommended as it demonstrates the arterial vascular supply, venous drainage, tracheobronchial tree and lung parenchyma.
- With the use of CT on the rise, we are likely to detect more cases in asymptomatic patients.
- Whilst it is important to identify and report these anomalies, given their potential implications, the decision on whether to offer treatment in asymptomatic patients should be deliberated.
